# Unlocking soil fertility: a physicochemical characterization of novel microalgal biofertilizers from *Tetradesmus nygaardii* and *Closteriopsis acicularis* for enhanced crop performance

**DOI:** 10.3389/fmicb.2026.1755868

**Published:** 2026-03-30

**Authors:** Anab Mujtaba, Rameesha Abid, Saira Bano, Tariq Khalil, Asif Jamal, Muhammad Zahid Qureshi, Muhammad Ishtiaq Ali

**Affiliations:** 1 Department of Microbiology, Quaid-i-Azam University, Islamabad, Pakistan; 2 Department of Environment and Natural Resources, College of Agriculture and Food, Qassim University, Buraidah, Saudi Arabia

**Keywords:** biofertilizer, *Closteriopsis acicularis*, sustainable agriculture, microalgae, plant physiology, sustainable crop production, *Tetradesmus nygaardii*

## Abstract

The transition toward sustainable agriculture has driven interest in microalgae as nutrient-rich biofertilizers. This study investigated the potential of two microalgal strains, *Tetradesmus nygaardii* (*T. nygaardii*), and *Closteriopsis acicularis* (*C*. *acicularis*), as biofertilizers for wheat (*Triticum aestivum*) and spinach (*Spinacia oleracea*). The microalgae were cultivated in a photobioreactor, and the resulting biomass was characterized using Fourier Transform Infrared Spectroscopy (FTIR), X-ray Diffraction (XRD), and Scanning Electron Microscopy (SEM). FTIR spectra confirmed the presence of functional groups such as amide band (C=O stretching of proteins) at peaks between 1640 and 1650 cm^−1^. XRD analysis indicated the presence of calcium carbonate (CaCO_3_), which supports root development in both crops. SEM analysis revealed a porous surface morphology ideal for nutrient retention and release. In field experiments, application of microalgal biomass, particularly the composite treatment of both strains with compost, significantly enhanced crop growth. This treatment increased chlorophyll content to 11.77 ± 0.33 mg/g in wheat and 14.41 ± 0.29 mg/g in spinach, representing substantial improvements over control values. Wheat plant height increased by 135% and grain yield reached 57.7 ± 8.57 per acre under the combined treatment, while spinach leaf count was doubled. Soil analysis confirmed enhanced fertility, with notable increases in nitrate and phosphate levels following biofertilizer application. This study demonstrates that microalgal biofertilizers, particularly the synergistic combination of *T. nygaardii* and *C*. *acicularis*, can improve crop productivity by up to 80% while enhancing soil health, representing a sustainable alternative to conventional fertilizers.

## Introduction

The growing world population of 2.5 billion in 1950 to more than 8 billion presently; the rising pace of industrialization and urbanization have exerted additional pressure on climate change, water shortage, and food security (Valavanidis, 2023; Zeng et al., 2025). These issues have undermined agricultural system, especially grain production with rising socioeconomic burden, globally (Sharma et al., 2025). Amidst these concerns, maintaining soil fertility and crop health particularly for low and middle-income countries like Pakistan are on the priority agenda of national policy (Hassan et al., 2025). During the last few decades, the exponential population growth in Pakistan has placed immense pressure on the government and agricultural sectors to modernize systems to mitigate food security risks. Besides having fertile lands of about 22 million hectares (Ahmed, 2024). Pakistan is rated lower than the countries with less airable lands causing a severe gap in actual and potential productivity. On the other hand, the productivity gap is further exacerbated by the present hike in the prices of synthetic fertilizers and increasing climate adversities, placing the future of 243 million people at high risk (Naz et al., 2025).

Currently, agriculture in Pakistan heavily relies on synthetic fertilizers rich in nitrogen, phosphorus, and potassium (NPK); however, the intensive application of these agro-chemicals poses significant environmental hazards including soil erosion, water contamination, disruption of microbial communities, and eutrophication (Khalili and Moridi, 2025). These challenges demonstrate the necessity to create low cost and environmental friendly solutions. Microbial biomass based biofertilizers are considered as the promising sustainable source of developing more robust agriculture ecosystem. Despite their environmental benefits, biofertilizers currently account for less than 2% of total fertilizer use in Pakistan (Singh et al., 2025).

The future of microalgae based biofertilizers has the potential to create sustainable agriculture since about 40% of world economic practices depend on agriculture (Kabato et al., 2025). Microalgae are a useful sources of nutrients, have the potential to support the functioning of biogeochemical processes, improve the nutritional content of soil, stimulate vegetation growth, and decrease the reliance on synthetic inputs that do not harm the environment (Sen et al., 2025). Microalgal strains such as *Chlorella vulgaris* (*C. vulgaris*) and *Spirulina platensis* (*S. platensis)* have shown better performance with a zero carbon footprint in field trials (Kiyani et al., 2025; Ummalyma et al., 2025). These strains possess high levels of bioactive compounds, which are phytohormones, polysaccharides, and antimicrobial substances, which can improve plant growth and quality of soil. Moreover, their ability to absorb waste streams in wastewater and sequester atmospheric carbon makes them potential new technologies to reduce environmental pollution and productivity limitation (Alrajeh et al., 2026).

Although studies have found that microalgae is a valuable and significant biological resource to achieve ecological sustainability, there is a knowledge gap in the effectiveness of indigenous strains. In particular, no prior study has defined the possible potential of *Tetradesmus nygaardii* (*T. nygaardii*) and *Closteriopsis acicularis* (*C. acicularis*) to use as a biostimulant for grain crops. Our hypothesis is that microalgae derived biofertilizers produced using *T. nygaardii* and *C. acicularis* will be highly effective in enhancing soil nutrient profile and improving the biometric parameters and yield in grain crops as they have unique bioactive compositions and can be used as a viable alternative to synthetic fertilizers. Thus, the current study was aimed at determining the appropriateness of the strains as nutrient rich biofertilizers. The results provide a cost effective, environmentally friendly, and workable solution to manage food safety and food security challenges for technologically developing countries like Pakistan.

## Methodology

### Acquisition of *C. acicularis* and *T. nygaardii* biomass

Two microalgal strains, *C. acicularis* (Accession ID: MT 858355.1) and *T. nygaardii* (Accession ID: MT 858750), previously isolated and identified in the Wastewater Treatment Lab at the Department of Biological Sciences, Quaid-i-Azam University, Islamabad, were evaluated for their potential application as biofertilizers. The microalgal strains were initially cultured in a 1 L glass bottle containing Bold's Basal Medium (BBM) under constant light and aeration. To prevent fungal contamination, Amoxil (1 mg/L), and Nystatin (1 ml/L) were added. BBM was sterilized through autoclaving at 121°C for 15 min. Purified microalgal cultures were confirmed microscopically using BBM agar plates. Subsequently, microalgae were grown in 10 L glass tanks containing domestic wastewater collected from Ramli village near Quaid-i-Azam University, Islamabad. Cultures were incubated for 21 days. After growth, the clear supernatant was collected for environmental analysis, and the algal biomass settled at the bottom was harvested at 60°C using a desiccator for 48 h and then ground into powder with a mortar and pestle.

### Establishment of the photobioreactor for the growth of microalgae

The design and construction of a laboratory scale photobioreactor (PBR) allowed for the controlled growing of microalgae in ideal environmental conditions. A clear cylindrical borosilicate glass vessel with a total working volume of 10 L made up the reactor. In order to ensure proper gas exchange and mixing, a sparger was coupled to an air pump that delivered sterile air enriched with 2% CO_2_ was used to provide continuous aeration. To provide even light distribution, LED lights and fluorescent tubes were placed around the reactor to provide illumination. To replicate normal diurnal conditions, the light intensity was kept at 120 μmol photons m^−2^ s^−1^ with a light: dark cycle of 16:8 h (Chen et al., 2011). A water jacket and thermostatic chamber were used to maintain the culture's temperature at 25 ± 2°C. To maintain the pH within the ideal range (6.5–7.0) for the chosen microalgae, 0.1 M NaOH and 0.1 M HCL were used. The pH was monitored and adjusted as needed.

To reduce contamination, all PBRs were kept under UV light for 20 min before inoculation. An exponentially growing culture of microalgae species added to the reactor along with sterile growth media (BG-11) at an initial cell density of 0.1 g/L dry weight or optical density (OD) at 680 nm. Chlorophyll content, biomass concentration and OD were measured at regular intervals to track the progress of the culture (Sharma, 2019).

### Evaluation of the effects of microalgal treatments on plant growth

Growth parameters, including biomass concentration, productivity and specific growth rate, were assessed. The OD was measured spectrophotometrically at 650 nm. Dry weight of biomass (mg/L) was recorded during the late exponential phase. Biomass productivity (g d^−1^ L^−1^) was calculated using the following formula:


$$\begin{array}{l} \text{Productivity }=\;\left({\text{Z}}_{2}-{\text{Z}}_{1}\right)/\left({\text{t}}_{2}-{\text{t}}_{1}\right) \end{array}$$

where Z_1_ and Z_2_ represent dry biomass at the start and end of the experiment. To determine the precise growth rate (g L^−1^ d^−1)^, the following equation was used.


$$\begin{array}{l} \text{Specific growth rate μ }=\text{ ln}{\text{N}}_{2}-\text{ln}{\text{N}}_{1}.\;{\left({\text{t}}_{2}-{\text{t}}_{1}\right)}^{-1} \end{array}$$

Here, N_1_ and N_2_ stand for the initial and final biomass concentrations, and (t_2_-t_1_)^−1^ stands for the time interval between these two measurements. This equation was used to compute generation time using the estimated specific growth rate.


$$\begin{array}{l} \text{Generation Time }\left(\text{T}\right)\;=\text{ ln}\left(2\right)/\text{μ} \end{array}$$

### Nutrient analysis and characterization of microalgal biomass as biofertilizer

The FTIR spectra was carried out at wavelengths of 4000–400 cm^−1^ using Parkin Elmer FTIR spectrometer (Waltham, USA) to detect chemical bonds and functional groups (proteins, carbohydrates, and lipids) that are important to soil fertility and plants growth. The measurement was done in Quaid-i-Azam University with the help of standard procedures (Manjunatha and Girisha, 2021). To characterize mineral composition and crystalline structure of microalgal biomass, X-Ray Diffraction (XRD) was employed, with special attention to such elements as Ca, Mg, Si, and P that influence nutrient availability and remain in the soil. The sample was analyzed at the Centralized Resource Laboratory, University of Peshawar through JEOL JDX-3532 XRD instrument. The microstructure and surface form of the dried microalgal biomass were examined under the Scanning Electron Microscopy (SEM). Preparations were made by mounting on carbon tape, gold sputter-coated and observed at 500x to 10,000x under a JEOL JSM5910 microscope at the University of Peshawar. Also, physicochemical characteristics of the microalgal strains were identified before the experiment (Table 1).

**Table 1:** Physiochemical properties of compost, compost tea and microalgal strains before the experiment.

S. No.	Parameters	Compost	Compost tea	*C. acicularis*	*T. nygaardii*
1	pH	7.20 ± 0.4	8.10 ± 0.66	6.7 ± 0.2	6.9 ± 0.4
2	EC dS/m	3.400 ± 0.25	2.81 ± 0.17	2.4 ± 0.5	2.4 ± 0.5
3	Organic matter %	32.52 ± .1	5.23 ± 0.37	74.6 ± 4.9	72.9 ± 4.7
4	C/N ratio	14.81 ± 0.2	10.67 ± 0.56	6.8 ± 1.6	6.9 ± 1.8
5	Total N%	1.370 ± 0.10	0.61 ± 0.04	5.7 ± 0.8	5.8 ± 0.9
6	Total P%	0.620 ± 0.05	0.18 ± 0.07	1.4 ± 0.9	1.6 ± 0.7
7	Total K%	0.850 ± 0.15	0.09 ± 0.02	1.8 ± 0.7	1.9 ± 0.8

### Seed pre-treatment and soil preparation

Certified seeds of wheat cultivar Borlaug 2016 were obtained from the National Agricultural Research Center (NARC), Islamabad, Pakistan. Seeds were disinfected with 95% ethanol and soaked overnight in distilled water before sowing. Soil was prepared by removing mechanical weeds, plowing, tillage, leveling, and loosening with a fork hole. Soil was sieved using a 2 mm strainer to remove clumps before sowing. Soil samples were collected randomly from each plot prior to sowing and after harvest. Physicochemical analysis, including pH and electrical conductivity (EC) was calculated by the digital multimeter (Lovibond SensoDirect 150), total available phosphorous and nitrate content were estimated by Olsen and Colorimetric method (Shinn, 1941; Sims, 2000) and organic matter was conducted to assess biofertilizer effects on soil health by following the protocol of Dkebska et al. (2016).

### Preparation of plant leaf and control

Plant leaf compost was prepared by vermicomposting fallen leaves under aerobic conditions at Quaid-i-Azam University. For this purpose, 500 g of mature compost was soaked in 10 L of tap water with continuous aeration for three days. The container was covered with a black plastic sheet to retain moisture, enhance microbial activity, regulate temperature and accelerate decomposition. Three teaspoons of unsulfured molasses were added to water to promote bacterial growth. Compost tea was applied as a foliar spray at 50 ml per pot every 2 weeks (Kim et al., 2015). Moreover, the physicochemical properties of compost and compost tea were also calculated before the experiment (Table 1).

### Experimental design and treatments

The field experiment was conducted at the experimental site of Quaid-i-Azam University, Islamabad (33.7293° N, 73.1317° E), from October 2024 to April 2025. Randomized Complete Block Design (RCBD) was used to control possible soil heterogeneity at the site. The 10 x 10 foot (100 sq.ft) experimental area was sub-divided into three main blocks, which represented three independent replicates (*n* = 3). Random assignment of plots in each block was done to maintain statistical validity. Eight treatment combinations were tested (Table 2) which included microalgal biomass (*C. acicularis* and *T. nygaardii*), organic compost and compost tea. Crops were cultivated under natural environmental conditions, and the same irrigation and weeding procedures were used in all the blocks to guarantee the validity of statistical comparisons.

**Table 2:** Experimental treatment combinations.

Sr. No.	Combinations	Compost (g/kg soil) and compost tea (ml/kg soil) dosage	Microalgae dosage (g/kg soil)
1	Control	-	-
2	Compost	10 g	-
3	*C. acicularis*	-	10 g
4	Compost + *C. acicularis*	5 g	5 g
5	*C. acicularis +* Compost tea	500 mL	5 g
6	*T. nygaardii*	-	10 g
7	Compost *+ T. nygaardii*	5 g	5 g
8	*T. nygaardii +* Compost Tea	500 mL	5 g
9	Compost + *C. acicularis+ T. nygaardii*	3 g	3.5 g + 3.5 g

### Soil analysis

Soil was analyzed before (Table 3) and after the experiment for pH, EC, assessment of total N, P, and K to determine nutrient deficiencies and soil fertility. Post harvest analysis of wheat and spinach soils monitored soil health indicators, nutrient depletion, and plan for future soil amendments (Dineshkumar et al., 2019).

**Table 3:** Physicochemical analysis of soil before plantation selected for experiment.

Characteristics	Soil
pH	8.29 ± 0.4
EC (μS/cm)	364
Available P (mg/kg)	4.1± 1.1
Available K (mg/kg)	96.5 ± 6.4
Available N (g/kg)	0.4 ± 0.2
Organic matter %	4.5 ± 2.2

### Physiological and morphological analysis of plant and chlorophyll estimation

Plant chlorophyll was extracted from 1 g fresh leaf tissue using 80% acetone, ground with magnesium carbonate, incubated at 4°C for 3 h, centrifuged at 2500 rpm for 5 min, and absorbance was measured at 645 nm and 663 nm using a spectrophotometer (SPAD 502 Plus) for total chlorophyll quantification (Kamble et al., 2015).

### Antioxidant enzyme assays

Enzyme extracts were prepared from 0.5 g leaf tissue ground in polyvinylpyrrolidone (PVP), EDTA, and 10 mL sodium phosphate buffer. Activities of superoxide dismutase (SOD), peroxidase and catalase were assayed spectrophotometrically following established protocols (Zhang and Kirkham, 1994).

### Morphological measurements

Pre- and post-harvest analysis included weekly monitoring of shoot and root length, fresh and dry weight, leaf count (spinach), grains per spike, grain weight per spike, total yield, and seed weight.

## Statistical analysis

The data was represented in the form of mean ± standard deviation. One-way Analysis of Variance (ANOVA) was used to determine the effectiveness of the various microalgal and compost treatments, which were carried out using IBM SPSS (version 26). The data analysis was preceded by the Shapiro-wilk test to verify the normality of data. Means difference of statistical significance was detected through the Tukey *post-hoc* test at a significant level of *p* < 0.05. Graphs were generated using Microsoft Excel.

### Results

**Figure 1:**
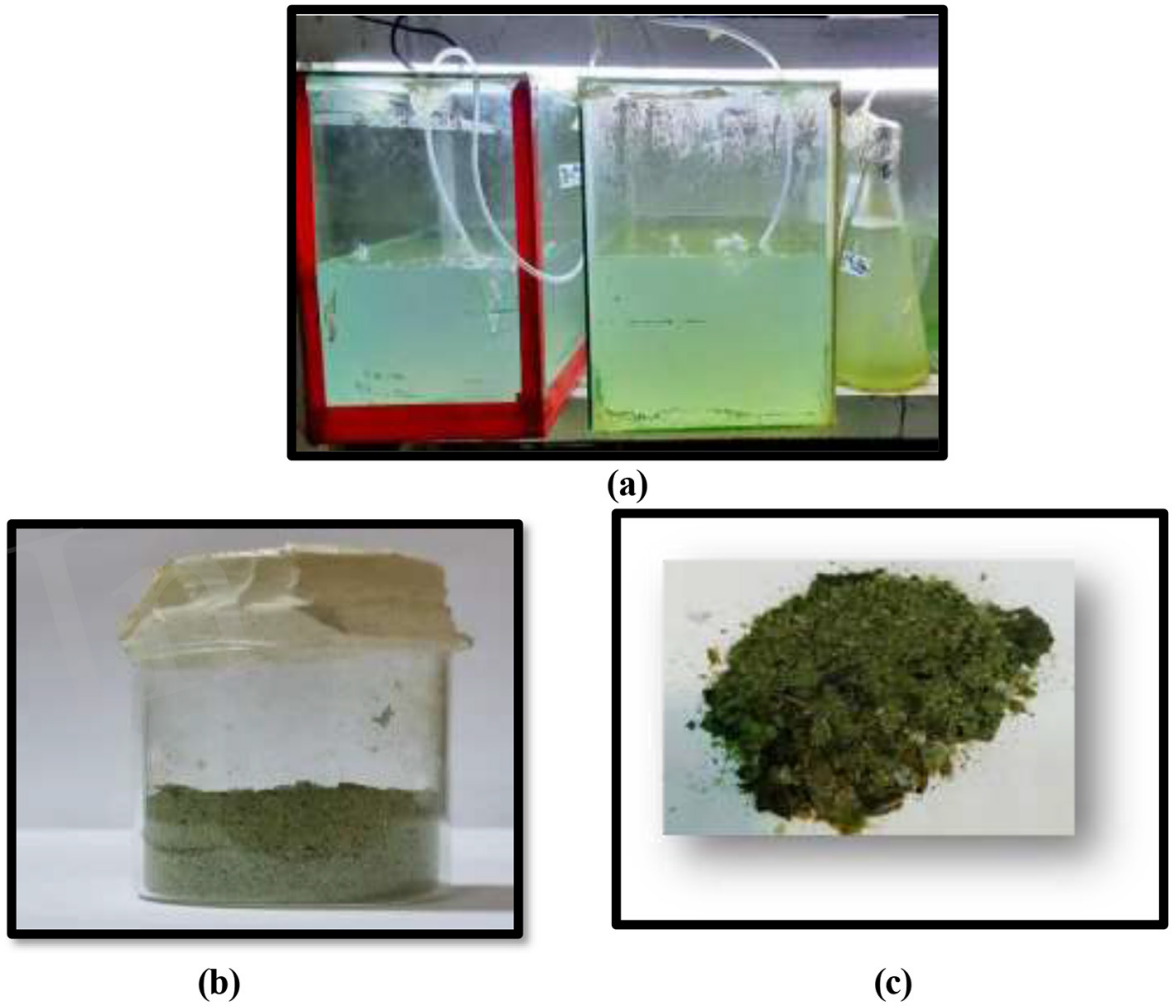
**(a)** PBR setup for microalgal strains *T. nygaardii* and *C. acicularis* **(b)** Cultivation and biomass harvested from strain *T. nygaardii* and **(c)** *C. acicularis*.

The PBR was set up in a greenhouse to facilitate the optimal growth of microalgal strains (Figure 1a). Parameters such as aeration, pH and light intensity were maintained within pre-determined optimal ranges to ensure steady cultivation conditions. Both the microalgal strains were harvested as well as desiccated on a 21st day cultivation stage (Figures 1b, c).

Growth monitoring was performed by measuring OD at 650 nm on days 3rd, 7th, 14th, and 21st to track microalgal growth (Table 4).

**Table 4:** OD measurements of *T. nygaardii* and *C. acicularis* microalgal strains.

No. of observations	Days	OD (AU cm^−1^) of strain *T. nygaardii*	OD (AU cm^−1^) of strain *C. acicularis*
1.	3	0.1201	0.1036
2.	7	0.5157	0.7971
3.	14	1.3349	1.4918
4.	21	1.3063	1.4123

## Characterization of microalgal biomass

**Figure 2:**
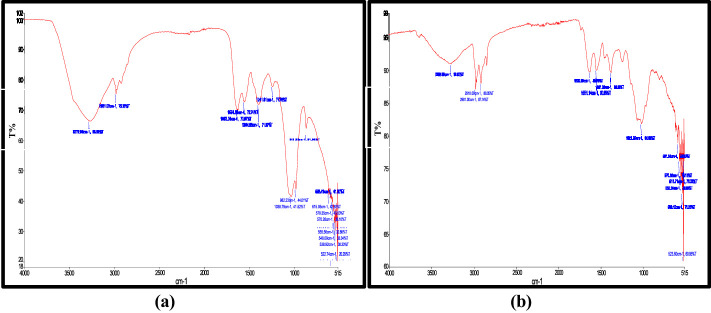
FTIR of microalgal strains **(a)** *T. nygaardii*, and **(b)** *C. acicularis*.

The analysis revealed characteristic absorption bands corresponding to biochemical components central to the biofertilizer potential of microalgae (Figure 2). The peaks were observed at 3,272.09 cm^−1^ (O-H, stretching of hydroxyl group), 2,920–2,850 cm^−1^ (C=C stretching of alkene) and 1,554.81 cm^−1^ **(**N-H stretching of secondary amines). Similarly, in Figure 2b, absorption peaks appeared at 1,394.98 cm^−1^ (CH_3_ stretching of tri-methyl groups), 1,038.79 cm^−1^ (P-O-C stretching of aliphatic phosphates) and 868.89 cm^−1^ **(**C-O-O stretching of ethers).

**Figure 3:**
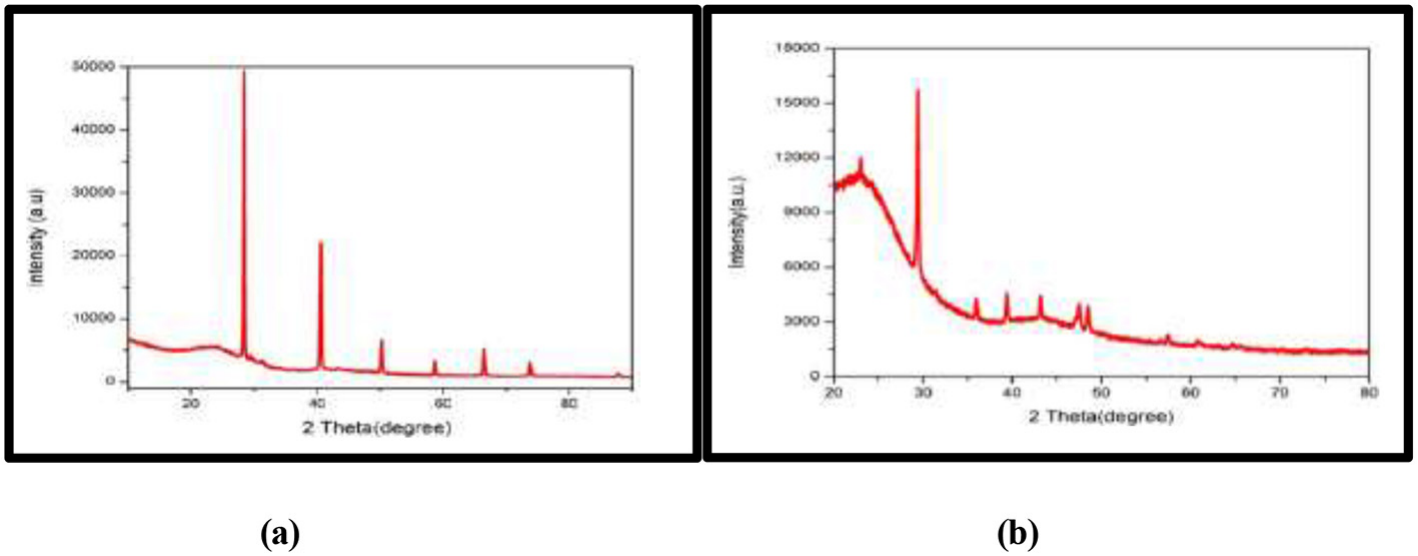
XRD spectrum of **(a)** *T. nygaardii* and **(b)** *C. acicularis*.

XRD analysis identified mineral components within the microalgal biomass that contribute to its biofertilizer properties. Sharp peaks observed in Figure 3a at 2θ values near 29.4°, 39.4°, and 47.5° correspond to calcium carbonate (CaCO_3_). In Figure 3b, magnesium compounds such as magnesium oxide or magnesium silicate were detected at 18.5°, 31.2°, and 45.1°. Additionally, a wide hump between 20° and 30° (2θ) was also observed in both the microalgal biomass.

**Figure 4:**
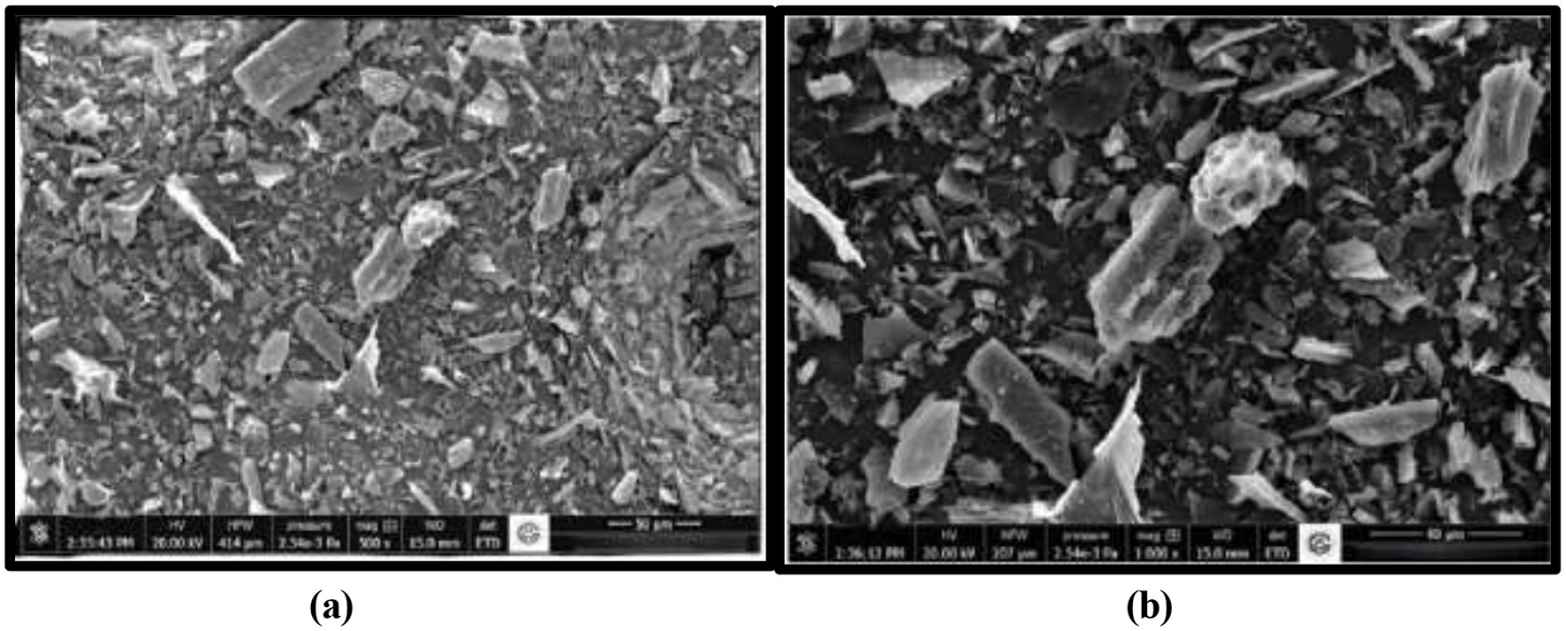
SEM images of **(a)** *T. nygaardii*, and **(b)** *C. acicularis*.

SEM imaging revealed that *T. nygaardii* has a rough, depression-like porous cell surface (Figure 4a). *C. acicularis* exhibited rod-shaped cells with an irregular surface and visible spore-like structures. Additionally, aggregation of biomass cells into micro-particles was observed (Figure 4b).

## Field scale experiment of wheat and spinach plant cultivation

**Figure 5:**
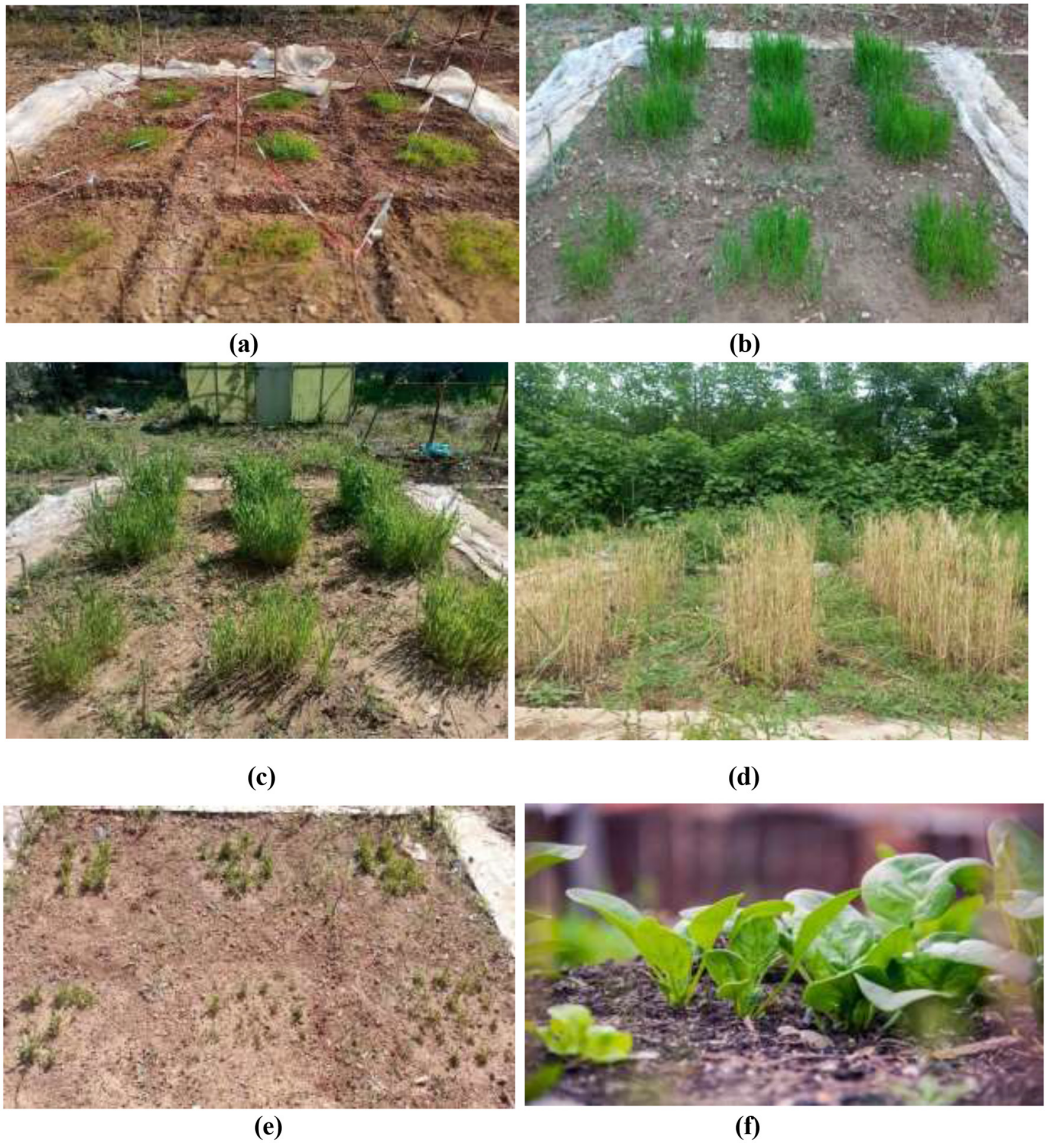
Field experimental setup showing wheat; **(a)** after 1 month tillering stage, **(b)** after 3-month stem elongation stage, **(c)** after 5-month heading stage, **(d)** after 6-month grain filling/mature stage. Spinach plant vegetative growth stages, **(e)** after 2 months, and **(f)** after 6 months of maturation stage.

Pictorial presentation of field scale experiment on different stages in 1–6 months of wheat and spinach plants (Figures 5a–f).

### Soil physicochemical analysis

Pre-harvest analysis of soil are represented in (Table 2) and soil post-harvest analysis, like N, P, K, pH, and EC, are depicted in (Table 5).

**Table 5:** Post-harvest soil analysis under different treatments.

Treatments	*N* (g/kg)	*P* (mg/kg)	*K* (mg/kg)	pH	EC (μs/cm)	Texture
Control	0.4 ± 0.6	4.1 ± 0.4	97 ± 9.6	7.26 ± 0.5	108.2 ± 1.3	Sandy loam
Compost	0.43 ± 0.4	5.6 ± 0.6	100.2 ± 1.6	7.2 ± 0.28	103.6 ± 1.42	Sandy loam
*C. acicularis*	0.45 ± 1.1	5.9 ± 0.3	101 ± 5.2	7.18 ± 0.59	95.5 ± 0.65	Sandy loam
Compost *+ C. acicularis*	0.46 ± 2.2	6.7 ± 0.4	104 ± 1.67	7.15 ± 0.33	96.7 ± 0.18	Sandy loam
*C. acicularis +* Compost tea	0.45 ± 0.4	6.1 ± 0.6	102.1 ± 5.4	7.01 ± 0.59	112.1 ± 0.59	Sandy loam
*T. nygaardii*	0.47 ± 1.5	6.3 ± 0.4	101.6 ± 8.2	7.06 ± 0.32	124.4 ± 0.41	Sandy loam
Compost *+ T. nygaardii*	0.49 ± 1.9	7.2 ± 0.44	106 ± 2.3	7.1 ± 0.21	101.9 ± 0.3	Sandy loam
*T. nygaardii +* Compost tea	0.44 ± 1.5	6.4 ± 0.5	102.5 ± 0.76	7.32 ± 0.44	118.5 ± 0.57	Sandy loam
Compost + *C. acicularis + T. nygaardii*	0.51 ± 2.8	7.5 ± 0.36	110 ± 3.05	6.97 ± 0.24	93.3 ± 0.3	Sandy loam

### Chlorophyll estimation

**Figure 6:**
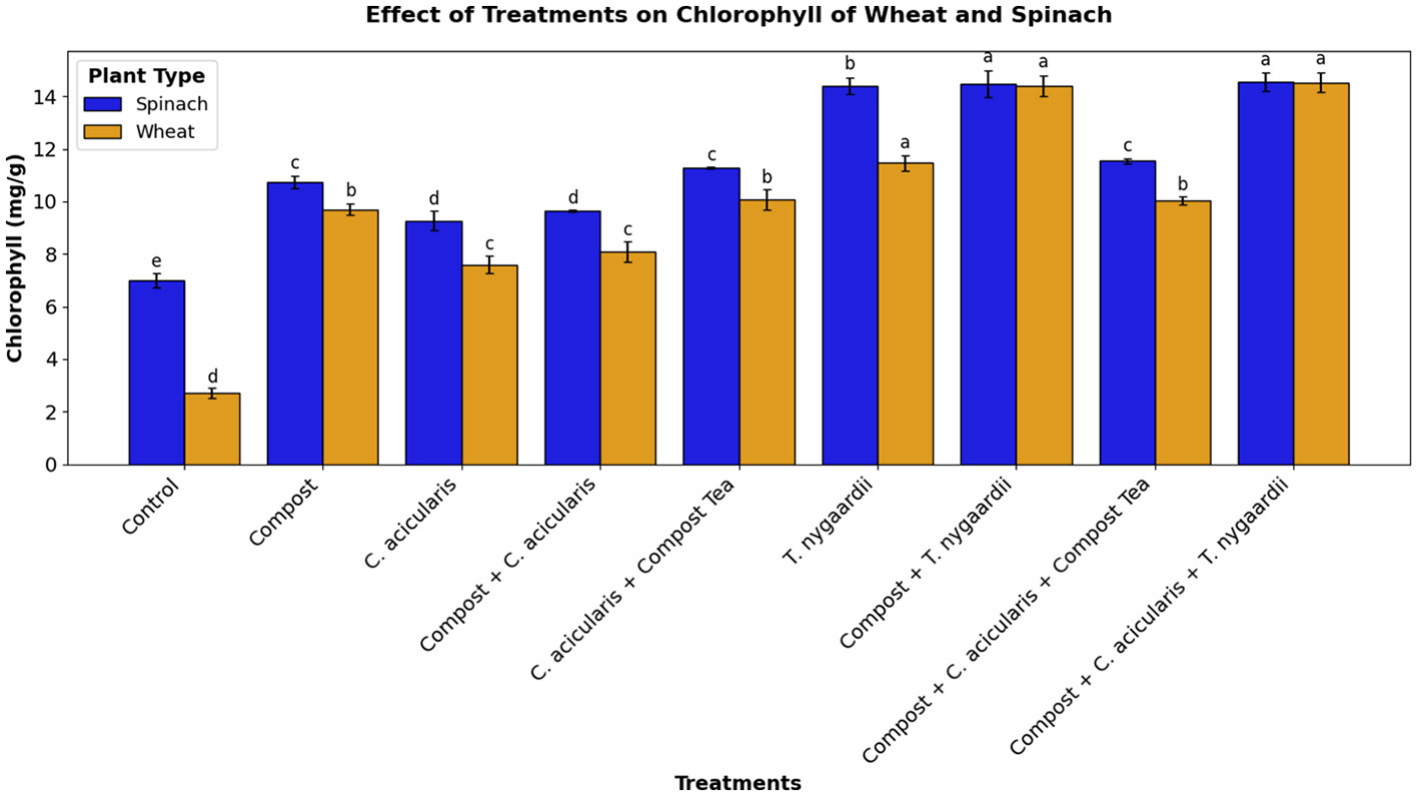
Chlorophyll content in wheat and spinach plants.

Chlorophyll content (mg/g) was measured for each treatment at 60, 90, and 120 days. The average total chlorophyll was calculated for each treatment and compared to the control. A significant correlation was observed among the different treatments. Chlorophyll levels in the control plants were markedly lower (2.71 ± 0.27 mg/g for wheat; 7.00 ± 0.20 mg/g for spinach) compared to the treatment groups. The highest chlorophyll content was recorded in Compost + *C. acicularis* + *T. nygaardii* group, reaching values as high as 11.77 ± 0.33 mg/g in wheat and 14.41 ± 0.29 mg/g in spinach (Figure 6).

### Plant antioxidant enzyme assays

**Figure 7:**
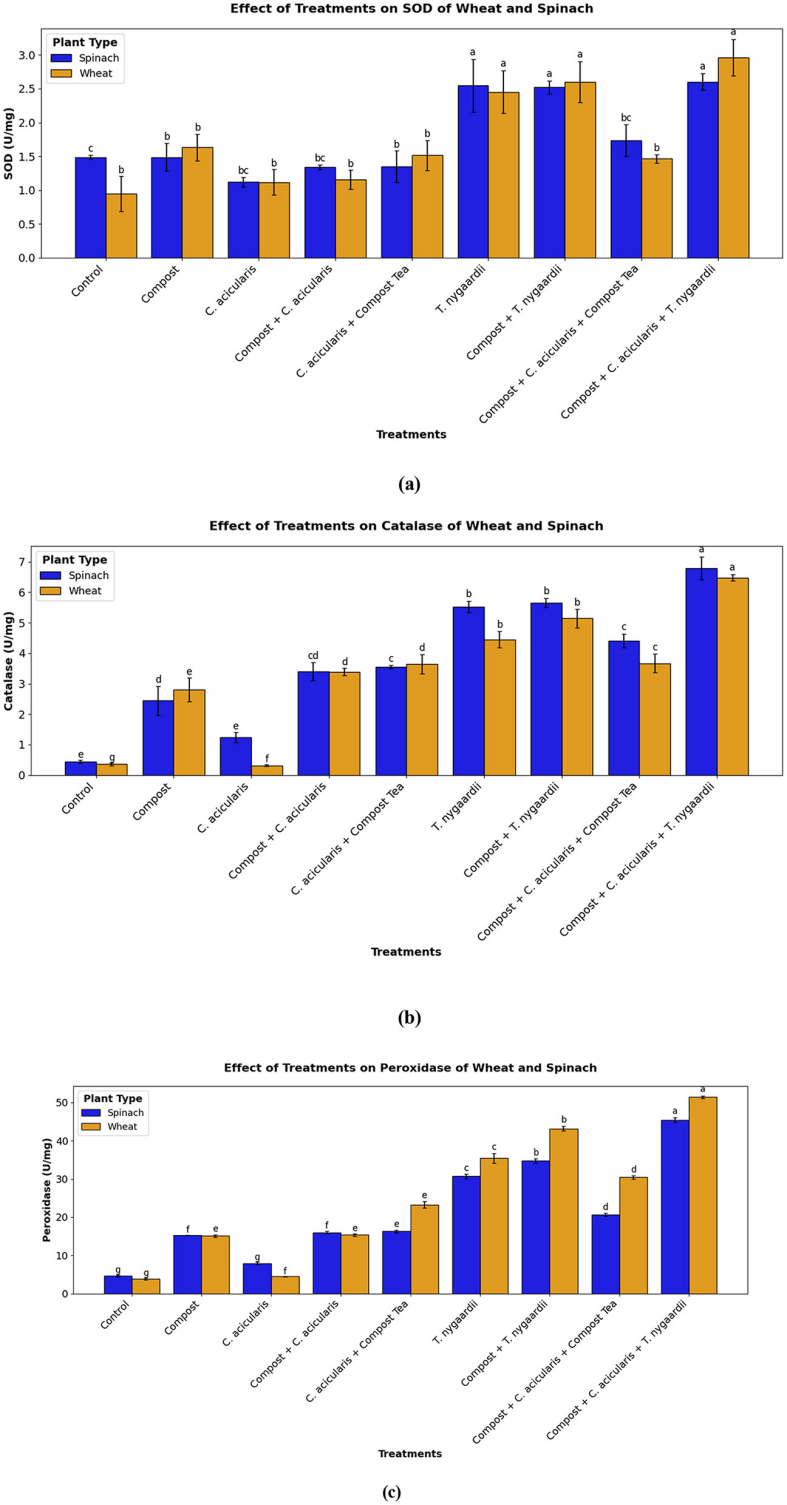
Plant antioxidant enzyme activities **(a)** SOD **(b)** Peroxidase **(c)** Catalase.

Biofertilizer treatments significantly (*p* < 0.001) enhanced SOD activity compared to the control group (0.95 ± 0.03 U/mg for wheat; 1.49 ± 0.26 U/mg for spinach). The highest SOD potential was recorded in plants treated with Compost + *C. acicularis* + *T. nygaardii*. Microalgae alone also induced a two-fold increase in antioxidant enzymes, indicating improved plant stress tolerance (Figure 7a). Catalase activity also showed significant increase in all the treated plants relative to the control (*p* < 0.001). While the control plants exhibited negligible activity (0.36–0.44 U/mg), the application of *T. nygaardii* and its combinations resulted in more than 10-fold increase in catalase activity. Specifically, the triple combination achieved the highest levels (4.15 ± 0.22 U/mg in wheat and 3.58 ± 0.17 in spinach; Figure 7b). Peroxidase activity exhibited the most significant variance among all parameters (*p* < 0.001). The untreated control showed the lowest activity (3.83 ± 0.25 U/mg for wheat and 4.62 ± 0.25 U/mg for spinach). In contrast the combined treatment of Compost + *C. acicularis* + *T. nygaardii* showed maximum activity reaching 23.82 ± 0.46 U/mg in wheat and 23.57 ± 0.41 U/mg in spinach (Figure 7c).

### Plant morphological analysis

**Figure 8:**
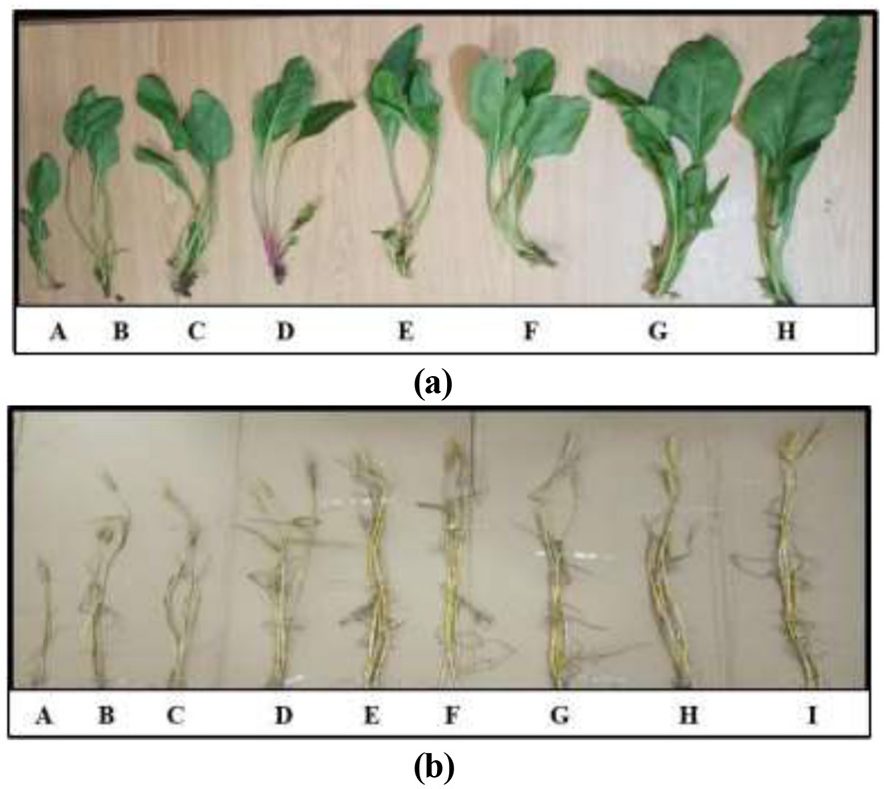
Plant morphological analysis with different combinations of microalgal strains; **(a)** Spinach (A) Compost (B) *C. acicularis* (C) Compost + *C. acicularis* (D) *C. acicularis* + Compost tea (E) *T. nygaardii* (F) Compost + *T*. *nygaardii* (G) *T. nygaardii* + Compost Tea (H) Compost + *C. acicularis* + *T. nygaardii*, **(b)** Wheat (A) Control (B) Compost (C) *C. acicularis* (D) Compost + *C. acicularis* (E) *C. acicularis* + Compost Tea (F) *T. nygaardii* (G) Compost + *T. nygaardii* (H) *T. nygaardii* + Compost Tea (I) Compost + *C. acicularis* + *T. nygaardii*.

Differences in shoot length with various combinations of *T. nygaardii* (Figures 8a) and *C. acicularis* (Figure 8b) on wheat and spinach plants.

**Figure 9:**
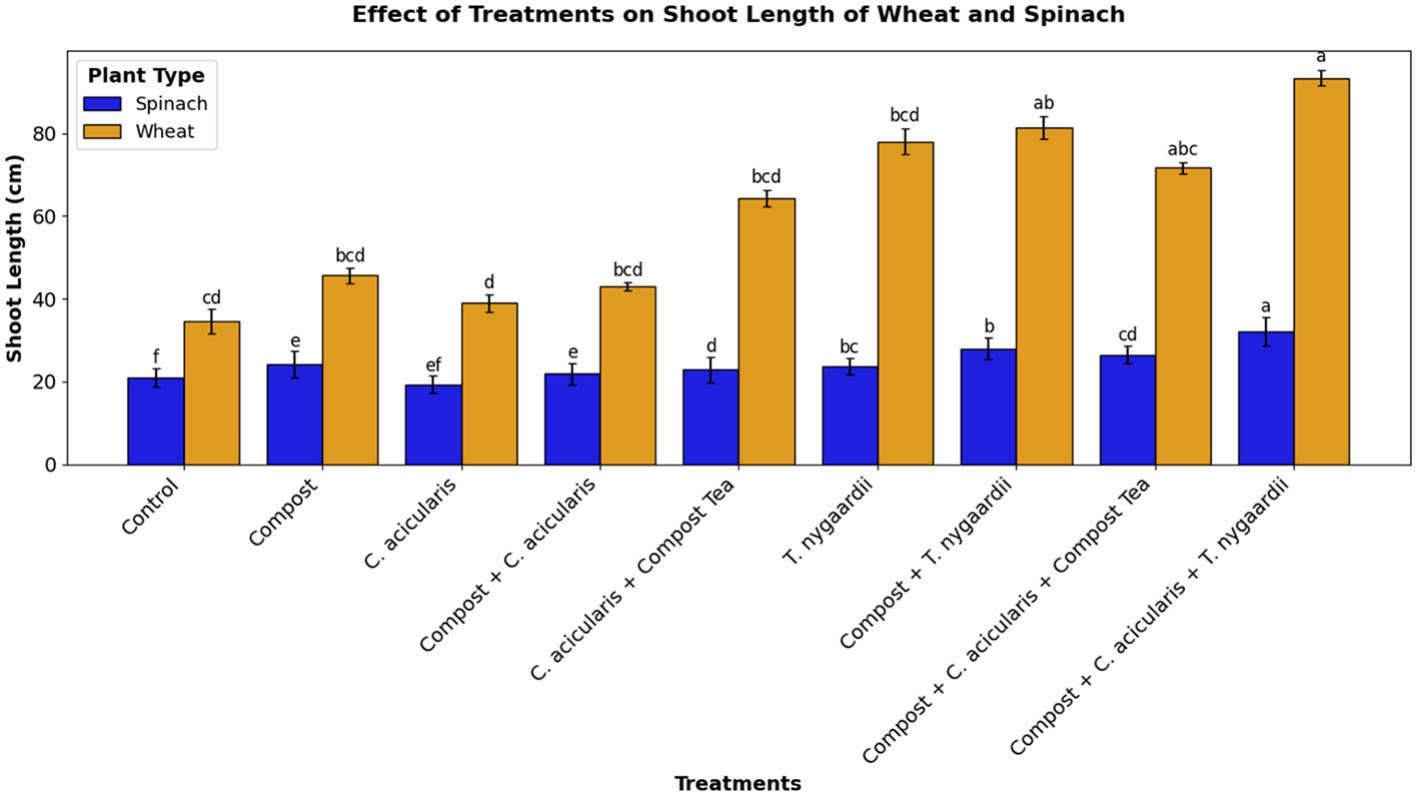
Shoot length of wheat and spinach crops under various treatments.

Significant increases in shoot length compared to the control (34.50 ± 2.29 cm for wheat and 21.00 ± 3.00 cm for spinach) were observed in treatments included *T. nygaardii* and *C. acicularis* and their combinations with compost. The Compost + *T. nygaardii* and Compost + *C. acicularis* treatments consistently showed the greatest shoot length. Final shoot length measurements at the time of harvesting during field scale experiment are shown in Figure 9.

**Figure 10:**
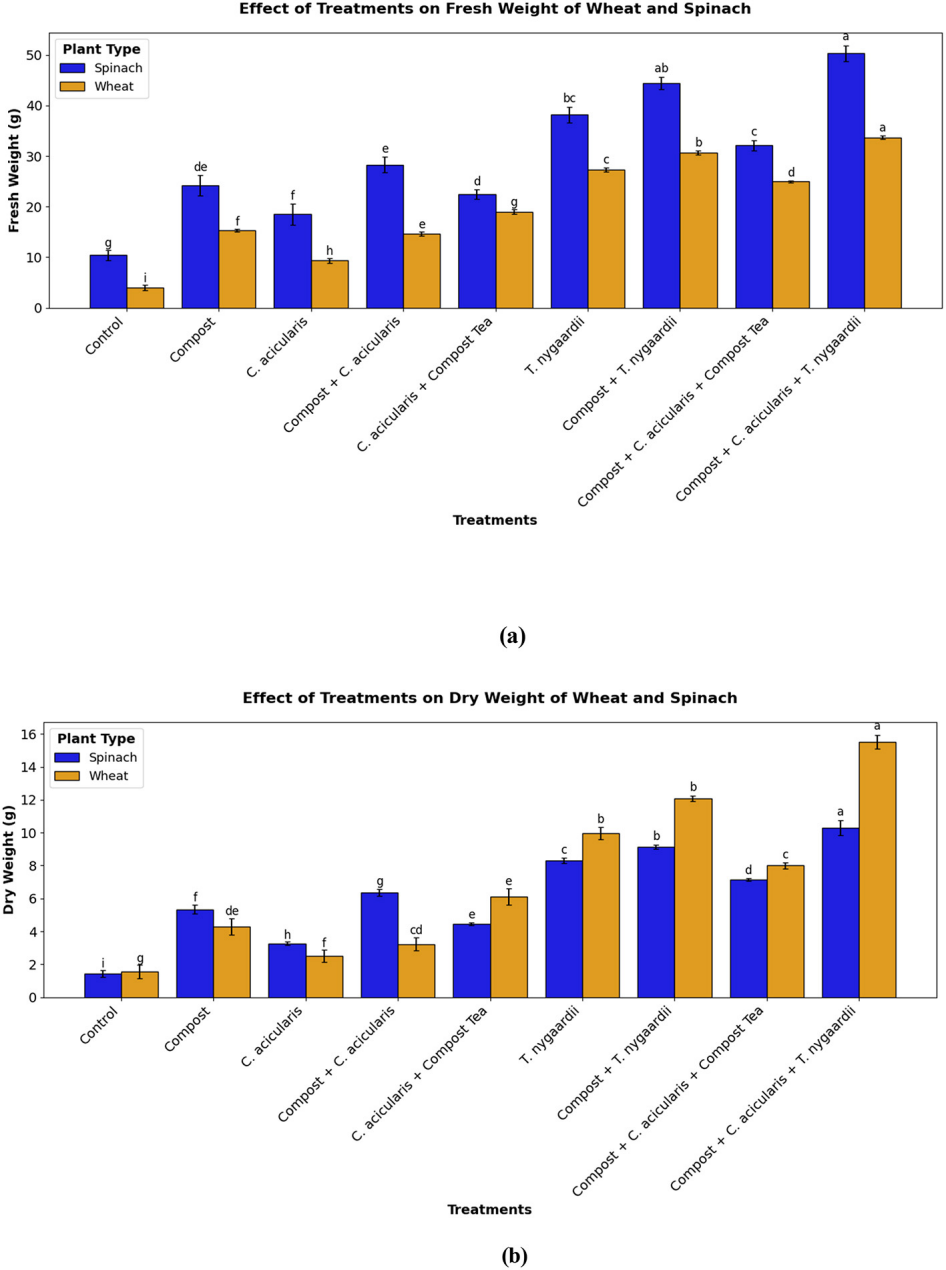
Fresh and Dry weight of edible crops at the maturity stage; **(a)** wheat crop, **(b)** spinach crop.

The cumulative biomass, represented by fresh weight, was significantly increased in the treatment groups (*p* < 0.001). The lowest fresh biomass was observed in control plants (4.00 ± 1.00 g for wheat and 10.44 ± 0.50 g for spinach). The most significant biomass accumulation was attained under the Compost + *C. acicularis* + *T. nygaardii* treatment group, where weights increased to 33.67 ± 1.53 g for wheat and 28.33 ± 0.35 g for spinach (Figure 10a). The control group observed significantly lower dry weights (1.57 ± 0.21 g and 1.43 ± 0.40 g for wheat and spinach respectively) compared to all treated groups (Figure 10b).

Wheat yield components, including grains per spike, total yield per acre, weight of grains per spike, and total seed weight, were highest in plants treated with a combination of compost + *C. acicularis* and *T. nygaardii* at a concentration of 10 g/kg soil (3 g compost + 3.5 g *C. acicularis* + 3.5 g *T. nygaardii*). All biofertilizer treatments, whether applied alone or in combination, showed significantly improved results compared to the control treatment. The control group produced the lowest yield 17.55 ± 1.67 yield/acre, which improved to 21.83 ± 0.97 yield/acre with the application of compost. The most superior results were found in the combined treatment of compost + *C. acicularis* and *T. nygaardii*, which achieved the maximum yield of 36.25 ± 1.32 yield/acre. Additionally, the analysis of the number of leaves per plant shows a clear upward trend in vegetative growth as treatments become more complex. The lowed leaf count was observed in the control group (7 ± 0.65), followed by *C. acicularis* (8 ± 0.73) and Compost + *C. acicularis* (10 ± 0.13). Productivity increased significantly with Compost (13 ± 0.47), while treatments involving *C. acicularis* + Compost tea (14 ± 0.53), *T. nygaardii* + Compost tea (14 ± 0.24) and *T. nygaardii* (15 ± 0.11) showed increased productivity. The highest results were achieved through Compost + *T. nygaardii* (15 ± 0.67) and the triple combination of Compost + *C. acicularis* + *T. nygaardii*, which peaked at 16 ± 0.53 leaves per plant.

### Discussion

The current study proves that the use of compost with *C. acicularis* and *T. nygaardii* has great potential as biofertilizer in the growth and yield of wheat and spinach plants. Previous studies on maize and onion by Dineshkumar et al. (2020) revealed similar findings, with increases in leaf number and plant height reported across all treatments over time. The gradual increase in OD values during the cultivation period showed active growth of microalgal cells, which ensured that the biomass applied was active in metabolism and enriched in primary metabolites (Sun et al., 2018; Table 4). In order to explain the mechanistic nature of these improvements, FTIR analysis of the microalgal biomass (Figure 2) indicated a suite of functional groups and biochemical components that are fundamental to biofertilizer use, such as typical peaks of 1,554.81 cm^−1^ (secondary amines) and 1,038.79 cm^−1^ (aliphatic phosphate; Arif et al., 2021). Several broad peaks indicated the presence of biologically active functional groups, including proteins, lipids, carbohydrates, organic acids, and phosphates, which act as key contributors to enhancing soil fertility and promoting plant growth. These bioactive groups are organic chelators and provide a slow-release process that prevents the rapid leaching of nitrogen and phosphorus into the environment, which in turn promotes the constant availability of these nutrients in the rhizosphere over a long period of time (Osorio-Reyes et al., 2023).

The biostimulant nature of the biomass was also evidenced by X-ray diffraction (XRD) analysis, which revealed mineral constituents that had a direct effect on the biofertilizer effect of the biomass (Chin et al., 2020). In Figure 3a, there were sharp diffraction peaks at 2θ values near 29.4°, 39.4°, and 47.5°, which corresponded to calcium carbonate (CaCO_3_) and it can be seen that the calcium minerals have the capacity to increase the soil pH and promote root growth, a fact supported by the considerably enhanced root lengths of the treated plants relative to the controls. Figure 3b showed that magnesium compounds (i.e., magnesium oxide or magnesium silicate) were found at 18.5°, 31.2°, and 45.1°, which may indicate the presence of micronutrients required to synthesize chlorophyll (Renuka et al., 2017). Magnesium (Mg) is a biologically essential element in the center of the chlorophyll porphyrin ring; its balanced release by the biofertilizer matrix directly promotes the synthesis of chlorophyll a and b, which in turn forms the major driving force of the observed rise in leaf greenness and photosynthetic capacity (Ahmed et al., 2023). The mineralogical support is surrounded with a wide hump at 20° and 30° (2θ) that indicates non-crystalline amorphous organic substances, such as proteins, lipids, and carbohydrates, which facilitate microbial activity and increase the proportion of organic matter in the soil. The algal biochar makes it easy to spread the macro- and micronutrients (C, O, Mg, Al, Si, Au, Nb, K, Ca, and Fe; Mona et al., 2024).

Besides the chemical characterization, SEM imaging showed that *T. nygaardii* possesses the rough, depression like porous cell surface that allows water and nutrient adsorption and facilitates gradual nutrient release when deposited on soil. Furthermore, this porous structure is also deep and therefore facilitates microbial colonization and hence increases soil microbial activity (Figure 4a). Conversely, *C. acicularis* had irregularly shaped cells whose surfaces appeared rod-like and had spore-like shapes, suggesting the existence of remnants of polysaccharides and organic matter, which contribute to the soil organic carbon (Figure 4b). Also, it was seen that the microalgal strains were able to aggregate biomass cells into micro-particles.

Efficacy of these structural and chemical characteristics was observed through the increased levels of N, P and K in the post-harvest analysis, which indicated the same level of efficacy as conventional chemical fertilizers (Table 5). In addition to nutrient composition, biofertilizer wastes enhanced the soil organic matter and fertility, water holding capacity, and beneficial microbial communities (Itelima et al., 2018). In our experiment, the enhanced soil conditions were the major factors that led to the rise in total biomass, as the triple combination (Compost + *C. acicularis* + *T. nygaardii*) produced the highest fresh and dry weights of the two microalgal species (Figures 10a, b).

The soil conditions were also improved, and this had a positive effect on plant antioxidant defense mechanisms. Regulated nutrient release as well as the introduction of microalgal biostimulants, altered the physiological condition of the plant leading to a stronger antioxidant enzyme reaction. Reactive Oxygen Species (ROS) are natural metabolites produced by plants during the intensive vegetative growth, and when they go unregulated, they can oxidatively damage cellular membranes (González-Pérez et al., 2022). Treatments of all biofertilizers promoted the production of SOD which implies that it can replace chemical fertilizers (Figure 7a). SOD is the primary biochemical defense of the plant against the superoxide radical (${\text{O}}_{2}^{-}$) in the transformation of ${\text{O}}_{2}^{-}$ into hydrogen peroxide (H_2_O_2_) (Ighodaro and Akinloye, 2018). As our findings confirm, it was a highly coordinated defense mechanism, in which the produced H_2_O_2_ was subsequently neutralized by considerably high concentrations of catalase and peroxidase (*p* < 0.001; Figures 7b, c). The observed rise in the peroxidase activity of the treated plants and specifically with the triple consortium group implies improved biochemical state of the plant, leading to cell-wall lignification and resistance to metabolic stress (Brito-Lopez et al., 2025).

**Figure 11:**
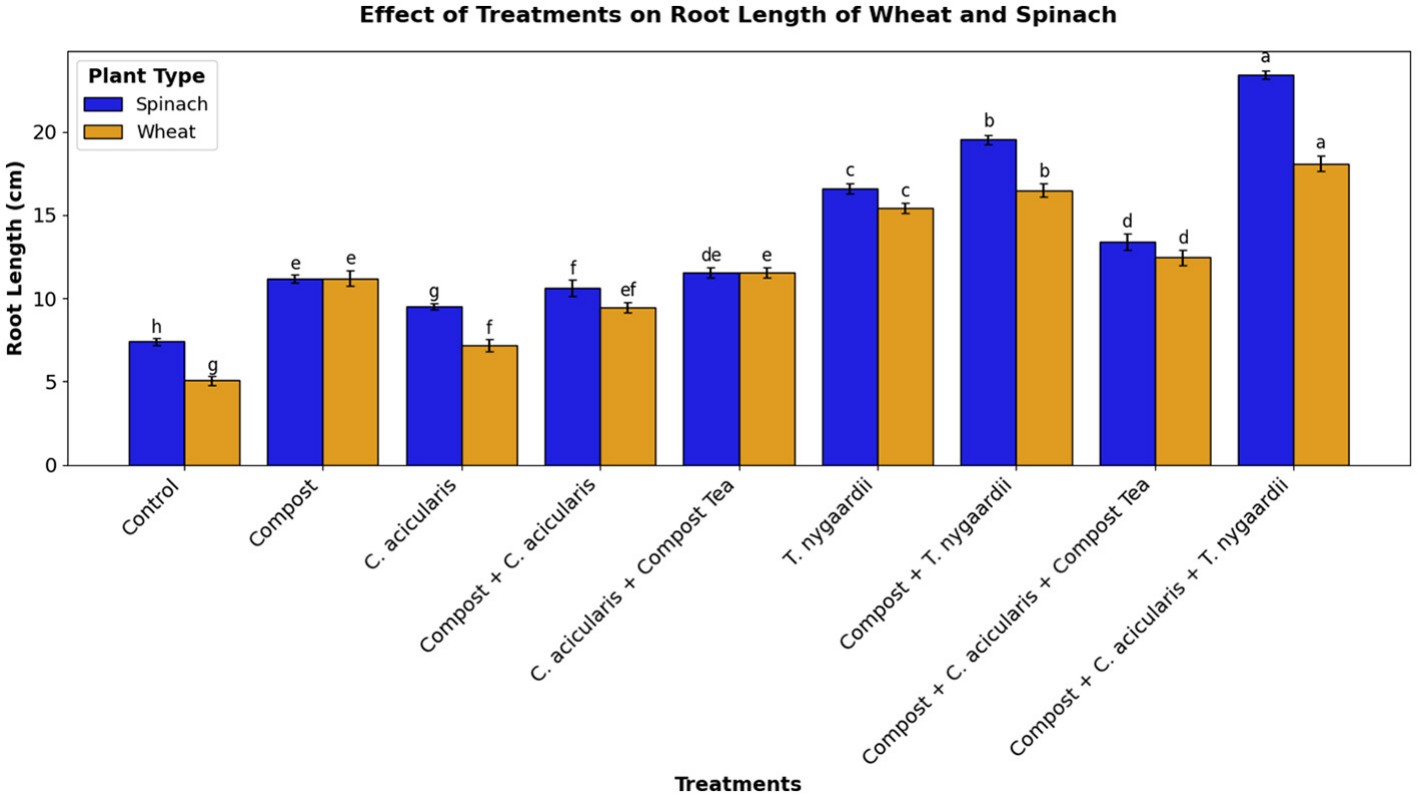
Root length of wheat and spinach plants under different treatments.

Notably, the synergistic effects between compost and microalgae showed strong biostimulant activity in plant yield and health (Day and Wiskich, 1995). Experiments on field scale demonstrated that biofertilizer treatments Compost + *C. acicularis* + *T. nygaardii*, Compost + *C. acicularis*, and Compost + *T. nygaardii* (applied at 10g/kg soil) gave superior results over control treatments. The positive effects of all the applications on the chlorophyll content of the plants were significant because of the enhanced nutrient availability (Figure 6). It is worth noting that the reduced level of fertilizer was more effective because plants have limited nutrient uptake potential (Ali et al., 2025). These increases in chlorophyll content were accompanied by greater vegetative growth, whereby the synergistic interaction between compost and microbes produced the highest shoot length and root proliferation (Figure 9 and Figure 11). This synergy resulted in the highest seed production and the number of leaves in spinach, which is consistent with previous research on chickpea and spinach with *C. vulgaris* (Ahamd et al., 2017). Nevertheless, despite such promising outcomes, microalgal biofertilizers are associated with several challenges such as high production costs, fluctuating nutrient levels, low rate of nutrient release, standardization, and competition with chemically stable agrochemicals (Miranda et al., 2024).

## Conclusion

This study concludes that *C. acicularis and T. nygaardii* are effective biofertilizers with substantial antioxidant potential, leading to increased peroxidase, catalase, and SOD enzymes. Physiological analysis showed that plants treated with Compost + *C. acicularis* + *T. nygaardii*, Compost + *C. acicularis* and Compost + *T. nygaardii* exhibited significantly higher total chlorophyll content. The lowest chlorophyll concentrations were obtained in control group and the application of compost by itself. The presence of phytohormones that contained N, P, and K in the microalgal powder was linked to the increase in chlorophyll levels of wheat. Morphological analysis revealed that the combined use of Compost + *C. acicularis* + *T. nygaardii* at the tested concentrations could favorably and positively promote the growth of wheat. Also, both *C. acicularis* and *T. nygaardii* have the potential to be developed further through genetic engineering which will enhance their biofertilizer efficacy on a variety of edible crops.

## Data Availability

The original contributions presented in the study are included in the article/supplementary material, further inquiries can be directed to the corresponding author/s.
