# Correction: Unlocking soil fertility: a physicochemical characterization of novel microalgal biofertilizers from *Tetradesmus nygaardii* and *Closteriopsis acicularis* for enhanced crop performance

**DOI:** 10.3389/fmicb.2026.1846358

**Published:** 2026-04-15

**Authors:** Anab Mujtaba, Rameesha Abid, Saira Bano, Tariq Khalil, Asif Jamal, Muhammad Zahid Qureshi, Muhammad Ishtiaq Ali

**Affiliations:** 1 Department of Microbiology, Quaid-i-Azam University, Islamabad, Pakistan; 2 Department of Environment and Natural Resources, College of Agriculture and Food, Qassim University, Buraidah, Saudi Arabia

**Keywords:** biofertilizer, *Closteriopsis acicularis*, sustainable agriculture, microalgae, plant physiology, sustainable crop production, *Tetradesmus nygaardii*

Affiliations of all authors as they appear in the published original version of the article (^1^ Department of Microbiology, Quaid-i-Azam University, Islamabad, Pakistan, ^2^ Department of Biochemistry, Qassim University, Buraidah, Saudi Arabia). Correcting affiliations (^1^ Department of Microbiology, Quaid-i-Azam University, Islamabad, Pakistan, ^2^ Department of Environment and Natural Resources, College of Agriculture and Food, Qassim University, Buraidah, Saudi Arabia).

In the published article, there was a mistake in the Keywords. The keywords were displayed as “biofertilizer, *Closteriopsis acicularis*, crop productivity, microalgae, plant growth, sustainable agriculture, *Tetradesmus nygaardii*”. The correct Keywords are “biofertilizer, *Closteriopsis acicularis*, sustainable agriculture, microalgae, plant physiology, sustainable crop production, *Tetradesmus nygaardii*”.

An incorrect **Funding** statement was provided. The correct funder is [Qassim University]/the correct **Funding** statement reads:

The author(s) declared that financial support was received for this work and/or its publication. This work was supported by Qassim University.

In the **Acknowledgments**, mistakenly added dual acknowledgents.

In the published article, there was a mistake in the **Acknowlegdments**. The **Acknowledgments** was displayed as “This research was funded by the Pakistan Science Foundation via the National Science Linkage Program (NSLP) under award number PSF/NSLP/C-QAU-895. We extend our appreciation to the deanship of Educational Services, Department of Biochemistry, Qassim University, Buraidah, Saudi Arabia.” The correct statement is “The researchers would like to thank the deanship of Scientific Research, Qassim University for funding the publication of this study”.

The original version of this article has been updated.

